# Short-Term Hypothermic Holding of Mouse Immature Testicular Tissue Does Not Alter the Expression of DNA Methyltransferases and Global DNA Methylation Level, Post-Organotypic Culture

**DOI:** 10.3389/fendo.2022.854297

**Published:** 2022-03-23

**Authors:** Riddhi K. Pandya, Shubhashree Uppangala, Sujith R. Salian, Sanjay Gupta, Guruprasad Kalthur, Stefan Schlatt, Satish Kumar Adiga

**Affiliations:** ^1^ Division of Clinical Embryology, Department of Reproductive Science, Kasturba Medical College, Manipal, Manipal Academy of Higher Education, Manipal, India; ^2^ Division of Reproductive Genetics, Department of Reproductive Science, Kasturba Medical College, Manipal, Manipal Academy of Higher Education, Manipal, India; ^3^ KS313, Epigenetics and Chromatin Biology Group, Advanced Centre for Treatment, Research and Education in Cancer, Tata Memorial Centre, Navi Mumbai, India; ^4^ Division of Reproductive Biology, Department of Reproductive Science, Kasturba Medical College, Manipal, Manipal Academy of Higher Education, Manipal, India; ^5^ Centre of Reproductive Medicine and Andrology (CeRA), University of Münster, Münster, Germany

**Keywords:** immature testicular tissue, organotypic culture, hypothermic holding temperature, DNA methylation, epigenetics, DNMT, fertility

## Abstract

**Introduction:**

Cryopreservation of immature-testicular-tissue (ITT) prior to gonadotoxic treatment, while experimental, is the only recommended option for fertility preservation in prepubertal boys. The handling and manipulation of ITT prior to banking could influence the functionality, genetic and epigenetic integrity of cells.

**Objectives:**

To investigate the impact of length of hypothermic holding of mouse ITT on the relative mRNA expression of the DNA methyltransferases (DNMTs) and global DNA methylation, post 14-days of organotypic culture.

**Methods:**

ITT from 6-day old mice were handled at hypothermic temperature (4 °C) for 6 and 24 h prior to 14-days organotypic culture. Relative mRNA expression of *Dnmt1, Dnmt3a*, and *Dnmt3b* along with global DNA methylation was measured from the cultured ITT.

**Results:**

No significant variation in the expression of *Dnmt1, Dnmt3a*, and *Dnmt3b* was observed in relation to varying holding time periods used. Further, global DNA methylation was comparable between 0, 6 and 24 h holding groups.

**Conclusions:**

Short-term holding of ITT at 4 °C does not affect the DNA methylation process post organotypic culture. While fully acknowledging the limitations of this approach in the mouse model, the results we presented in this report will be of significant interest to the field.

## Introduction

Childhood cancer survivors are at risk of experiencing infertility as one of the long-term health complications of cancer therapy. In males, this is due to the potential impact of gonadoxic agents on actively dividing spermatogonial stem cells (SSCs), which provide the foundation for normal spermatogenesis (1–4). To overcome this health hazard, currently, cryopreservation of immature testicular tissue (ITT) is the only available fertility preservation option for prepubertal boys as spermatogenesis is not fully functional in them. However, this approach is still considered experimental (5, 6).

Due to the limited number of centers that offer ITT banking worldwide (5), transporting the tissue from the testicular biopsy site to the tissue banking facility is inevitable. In this regard, studies have determined the optimal conditions for ITT such as tissue size, storage temperatures, and storage periods in various models including human tissue (7–11). Recently, our group has demonstrated that ITT manipulation at 4°C had a minimal negative impact on the organotypically cultured germ cell population when compared to room temperature and 37°C (12). However, we believe that it is important to address the epigenetic integrity of germ cells from cultured ITT as epigenetic aberrations may negatively affect the subsequent developmental process.

DNA methylation is critical for fertilization, embryonic development, and postnatal life (13–17). The family of DNA methyltransferases (DNMT’s) consists of *Dnmt1*, *Dnmt3a*, and *Dnmt3b*, responsible for maintenance and *de novo* establishment of methylation patterns on 5’-positions of cytosine on DNA (18, 19). Epigenetic modifications are heritable changes in gene function independent of alterations in DNA sequence (20, 21). Although studies have shown that global DNA methylation level and expression of enzymes responsible for DNA methylation are unaffected during *in vitro* culture (22, 23), the impact of hypothermic holding of ITT prior to the organotypic culture on DNMT’s expression and global DNA methylation is not elucidated so far. Hence, using the mouse model, this study was aimed to investigate the impact of hypothermic holding of ITT on the relative mRNA expression of the DNMT’s and global DNA methylation post-14-days of organotypic culture.

## Materials and Methods

### Animals, Ethical Clearance, and Testicular Tissue Collection

A total of twenty-four, 6 day-postpartum (dpp) male Swiss albino mice were used in the study. All experiments and animal handling were conducted in accordance with the institutional guidelines for animal experimentation after obtaining prior approval from the Institutional Animal Ethics Committee (Kasturba Medical College & Kasturba Hospital Institutional Ethics Committee, approval #IAEC/KMC/93/2013). Animals were sacrificed by cervical dislocation and the testes were collected in alpha minimum essential medium (α-MEM + Glutamax; (32571-036; Gibco™, Grand Island, USA) containing 1% (v/v) penicillin-streptomycin (Pen-Strep; 15140-122; Gibco™, Grand Island, USA) and 5 µg/mL Nystatin (Nys; N3503; Sigma-Aldrich, St. Louis, USA). Testes were made fat-free using fine needles, under the stereomicroscope, and later randomly distributed/categorized for either holding-phase or direct culture.

### Holding Phase of Testes

**Figure 1:**
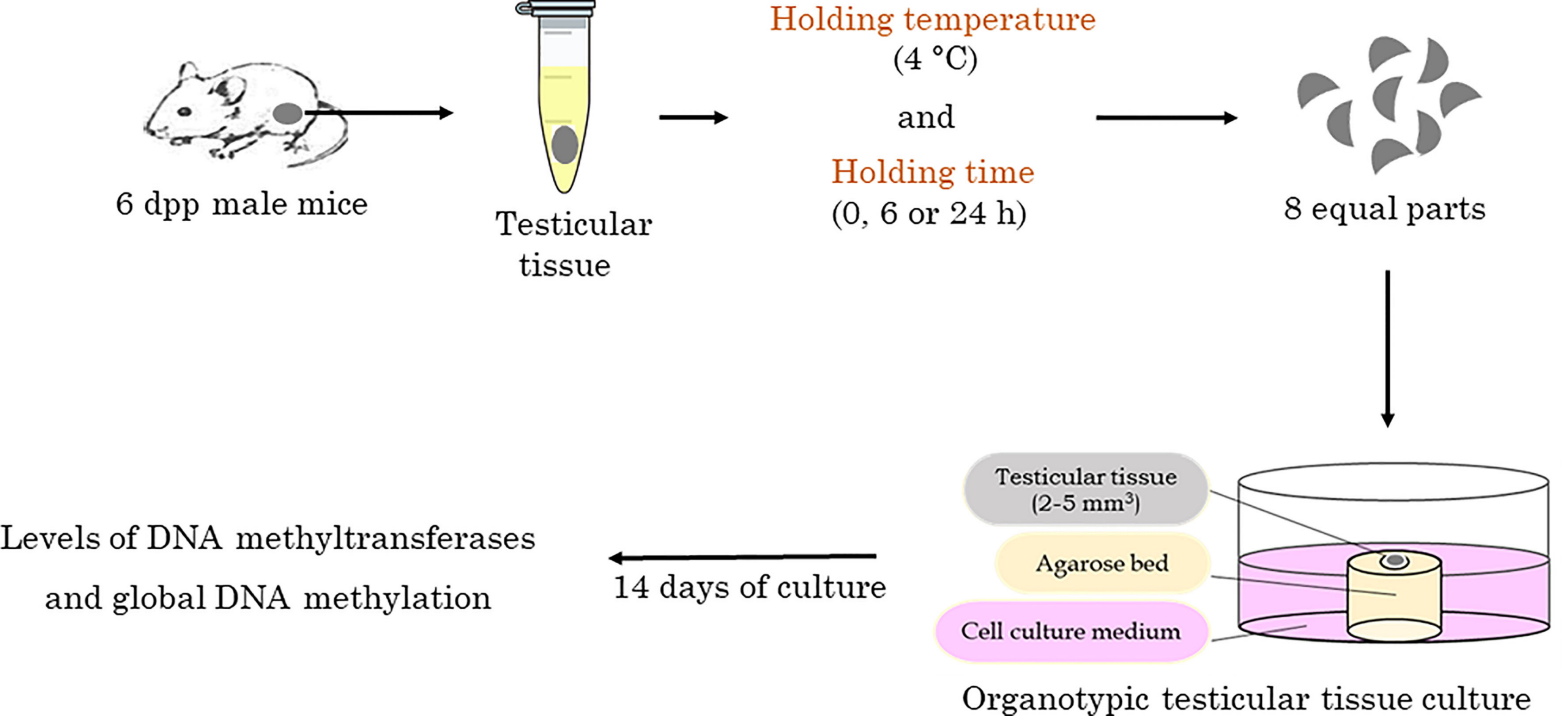
Flowchart of the study. Experimental outline to assess the impact of short-term hypothermic holding on epigenetic integrity of the testicular tissue.

Holding the 6 dpp testes at hypothermic temperature (~4°C) was performed as depicted in the experimental outline (**Figure 1**). Briefly, the excised 6 dpp testes were cultured directly or transferred to tubes containing α-MEM + Glutamax media supplemented with 10% knock-out serum replacement (KSR; 10828-010; Gibco™, Grand Island, USA), with Pen-Strep and Nys, using a sterile forceps. The tubes were placed in hypothermic, i.e., in a cooling unit maintained at ~4°C. The holding phase interval was scheduled as 6 and 24 h, corresponding to short-range and long-range shipment. Post holding, these testes were processed for organotypic culture as described previously (12).

### Isolation of Total RNA, cDNA Synthesis, and Gene Expression Analysis

Total RNA was extracted from ITT using TRIzol reagent (15596018, Ambion life technologies, USA). 1 µg of total RNA was reverse transcribed using random primers by a high-capacity cDNA RT kit (4368814, Applied biosystems, USA) according to the manufacturer`s protocol.

Quantitative polymerase chain reaction (*q*PCR) was carried out using Premix Ex Taq kit (RR390A, TaKaRa Bio, Japan), in StepOne™ Real-Time PCR System (Thermo Fisher Scientific, USA). TaqMan assay (Thermo fisher scientific, USA) for DNA methyltransferases viz. *Dnmt1* (Mm01151063_m), *Dnmt3a* (Mm00432881_m) and *Dnmt3b* (Mm01240113_m1) were used. *q*PCR results were normalized to *Actb* and *Gapdh* reference genes.

### DNA Extraction and Global DNA Methylation Analysis

DNA was extracted from 25 mg of cultured ITT using QIAamp DNA Mini Kit (51306, Qiagen, CA, USA) according to the manufacturer`s protocol. Extracted DNA samples were eluted with 100 µl of TE buffer and stored at -20°C until further needed. The global DNA methylation was measured using MethylFlash™ Methylated DNA Kit (P-1034-96, Epigentek, NY, USA) according to the manufacturer’s instructions. Briefly, methylated DNA was detected by 5-methyl cytosine (5-mC) antibody and quantified by colorimetric absorbance method at 450 nm using Multiskan™ FC Microplate Photometer (51119000, Thermo fisher scientific, Massachusetts, USA). The amount of methylated DNA was proportional to the OD intensity measured. Percent global DNA methylation (%5-mC) was calculated from the OD by the generated standard curve.

### Statistical Analysis

Data were expressed as Mean ± SEM. The data were analyzed for normal distribution by the Shapiro-Wilk test. All the parameters were analyzed by one-way analysis of variance (ANOVA) using GraphPad Prism 8 (GraphPad Prism software, CA, USA). The data were considered significant at p < 0.05.

## Results

### Effect of Holding the ITT on mRNA Expression of DNA Methyltransferases

**Figure 2:**
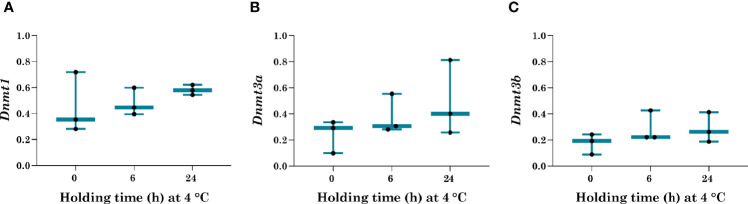
mRNA expression of *Dnmt1*, *Dnmt3a*, and *Dnmt3b*. Real-time *q*PCR analysis to understand the effect of varying holding duration at 4°C on mRNA levels of **(A)** *Dnmt1*, **(B)** *Dnmt3a*, and **(C)** *Dnmt3b*. ITT held for 0 h at 4°C cultured for 14 days was used as a control in comparison to 6 and 24 h held cultured ITT in similar conditions. mRNA level of *Dnmt1*, *Dnmt3a*, and *Dnmt3b* gene was normalized against reference genes *Actb* and *Gapdh.* Data are presented as Mean **±** SEM (n = 3).

To assess the effect of ITT holding at 4°C for varying duration on the mRNA expression of DNA methyltransferases, *Dnmt1*, *Dnmt3a*, and *Dnmt3b* was analyzed using real-time *q*PCR. The relative expression of all three genes was normalized against the 6 dpp control group. The relative expression of maintenance DNA methyltransferases, *Dnmt1* was comparable in all the holding groups (**Figure 2A**). Further, the levels of mRNA transcripts of *de-novo* methyltransferases, *Dnmt3a* and *Dnmt3b* did not vary significantly in post-organotypic cultured ITT held at 4°C for various time periods (**Figures 2B, C**). Lack of statistical significance could be attributed to the variations in Ct values of real time PCR.

### Effect of ITT Holding Prior to Organotypic Culture on Global DNA Methylation

**Figure 3:**
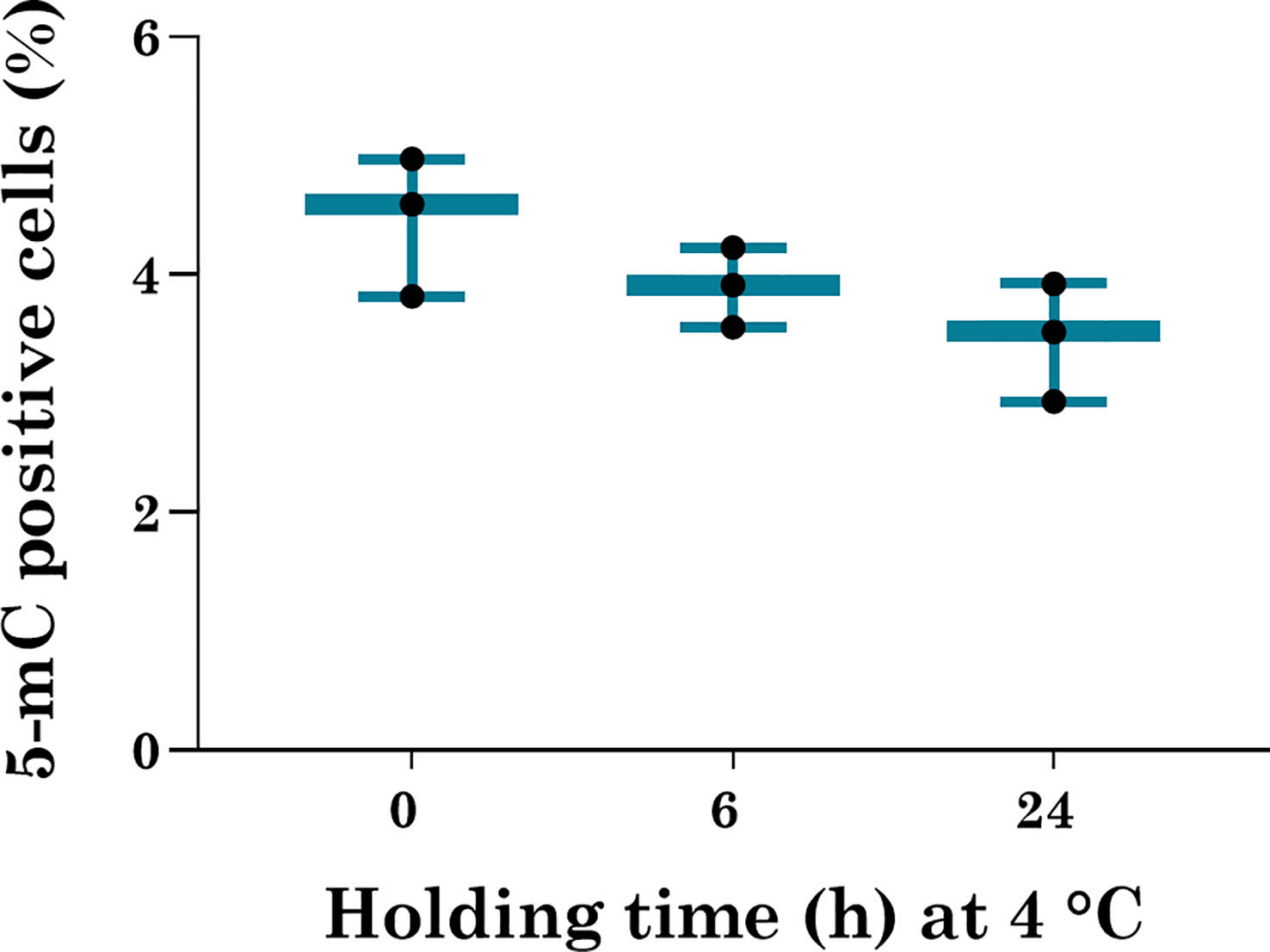
Global DNA methylation analysis in cultured ITT. Levels of percent methylated cytosine (5-mC) in cultured ITT. Cultured ITT held at 0 h intervals were used as a control in comparison to cultured ITT held for 6 and 24 h at 4°C. The data is presented in mean ± SEM (n = 3).

5-methylcytosine (%5-mC) level was analyzed to explore the impact of holding temperature and length of holding on global DNA methylation. Though a moderate decline in %5-mC level was observed in 6 and 24 h holding time in comparison to 0 h, the differences were not statistically significant (**Figure 3**). This observation indicates that short-term hypothermic storage of ITT does not alter the global DNA methylation level post-organotypic culture.

## Discussion

The correct establishment of DNA methylation in developing germ cells depends on DNMT expression. The results from this study have demonstrated that short-term hypothermic holding of mouse ITT up to 24 h has no significant impact on the expression of DNMTs and global DNA methylation in organotypic cultured ITTs.

Previous studies have emphasized the importance of conventional DNA methylation during male germ cell development. DNA methyltransferases have dynamic expression during the proliferation and differentiation phase of spermatogenesis (24, 25). Targeted deletion of *de-novo* methyltransferases in prenatal male germ cells showed lower levels of DNA methylation in postnatal spermatogonia. Also, spermatogenic arrest and infertility were observed in such methyltransferases, deficient mouse models (24, 26, 27).

Therefore, in this study, we investigated the expression of DNMTs in cultured ITT after the tissue was subjected to hypothermic holding up to 24 h. The relative expression of *Dnmt1*, *Dnmt3a*, and *Dnmt3b* did not vary significantly between the varying holding time periods tested. This observation is in agreement with previous reports where the expression patterns of DNMT’s were unchanged in *in vitro* and *in vivo* derived spermatozoa (22, 23, 28). It has been also shown that fresh and frozen-thawed ITT can maintain DNMT1 and DNMT3A expression even up to 30 days of *in vitro* culture (23). Furthermore, spermatogonial stem cells obtained from non-human primates could maintain DNMT expression during short-term culture *in vitro* (22). Our data add new information to the existing literature that holding ITT up to 24 h at 4 °C has a minimal adverse effect on the DNA methylation process. Nevertheless, the Ct value variations in three trails could have affected the level of statistical significance. Hence, observations made in this study should be considered with caution.

The establishment of global DNA methylation in spermatogonial stem cells plays a key role in spermatogonial identity, its differentiation potential, and the accurate transmission of epigenetic information to the next generation (14, 15, 29). Most of the studies examining the global DNA methylation level in mouse testis were found to be stable at the postnatal period (29, 30). Also, Spermatozoa produced from fresh/cryopreserved *in-vitro* matured ITT had un-fragmented and condensed nuclear DNA (31).

Hence, it is important to understand the impact of ITT manipulation on the global DNA methylation level as fertility preservation techniques can coincide with the window of the establishment of global DNA methylation. Our observation showed hypothermic holding of ITT at 4°C for 24 h could decrease the global DNA methylation level (%5-mC) moderately, though it is not possible to establish the statistical significance in our study. Earlier, it has been shown that sperm derived from frozen-thawed ITT had a similar intensity of 5-mC compared to sperm derived *in vitro* (23).

While fully acknowledging the limitations of this approach in the mouse model, we feel that the results we presented in this report will be of significant interest to the field. We show that short-term holding of ITT at 4 °C does not affect the DNA methylation process. However, future research should focus on addressing the methylation errors in specific imprinted genes in human prepubertal tissues.

## Data Availability Statement

The original contributions presented in the study are included in the article/supplementary material. Further inquiries can be directed to the corresponding authors.

## Ethics Statement

The animal study was reviewed and approved by Kasturba Medical College & Kasturba Hospital Institutional Ethics Committee, approval #IAEC/KMC/93/2013.

## Author Contributions

Conceived and designed the experiments: SA. Performed the experiments and was involved in the acquisition of data: RP and SRS. Analyzed and interpreted the data: RP and SU. Wrote the manuscript: SKA, SS, RP, and SU. Revised the manuscript critically for important intellectual content: GK and SG. RP is the guarantor of this work and as such, had full access to all the data and takes responsibility for the integrity of the data and the accuracy of the data analysis. All authors have given final approval for publication.

## Funding

This work was supported by the research grants from the Indian Council of Medical Research (ICMR # 5/10/FR/8/2014-RCH) and Science and Engineering Research Board (SERB) research grant (EMR/2015/000012).

## Conflict of Interest

The authors declare that the research was conducted in the absence of any commercial or financial relationships that could be construed as a potential conflict of interest.

## Publisher’s Note

All claims expressed in this article are solely those of the authors and do not necessarily represent those of their affiliated organizations, or those of the publisher, the editors and the reviewers. Any product that may be evaluated in this article, or claim that may be made by its manufacturer, is not guaranteed or endorsed by the publisher.
